# *Stenotrophomonas maltophilia* PhoP, a Two-Component Response Regulator, Involved in Antimicrobial Susceptibilities

**DOI:** 10.1371/journal.pone.0153753

**Published:** 2016-05-09

**Authors:** Ming-Che Liu, Yi-Lin Tsai, Yi-Wei Huang, Hsing-Yu Chen, Po-Ren Hsueh, Szu-Yu Lai, Li-Chia Chen, Yi-Hwa Chou, Wen-Yuan Lin, Shwu-Jen Liaw

**Affiliations:** 1 Department and Graduate Institute of Clinical Laboratory Sciences and Medical Biotechnology, College of Medicine, National Taiwan University, Taipei, Taiwan, Republic of China; 2 Department of Biotechnology and Laboratory Science in Medicine, National Yang-Ming University, Taipei, Taiwan, Republic of China; 3 Department of Clinical Pathology, Taipei City Hospital Renai Branch, Taipei, Taiwan, Republic of China; 4 Department of Laboratory Medicine, National Taiwan University Hospital, Taipei, Taiwan, Republic of China; University Paris South, FRANCE

## Abstract

*Stenotrophomonas maltophilia*, a gram-negative bacterium, has increasingly emerged as an important nosocomial pathogen. It is well-known for resistance to a variety of antimicrobial agents including cationic antimicrobial polypeptides (CAPs). Resistance to polymyxin B, a kind of CAPs, is known to be controlled by the two-component system PhoPQ. To unravel the role of PhoPQ in polymyxin B resistance of *S*. *maltophilia*, a *phoP* mutant was constructed. We found MICs of polymyxin B, chloramphenicol, ampicillin, gentamicin, kanamycin, streptomycin and spectinomycin decreased 2–64 fold in the *phoP* mutant. Complementation of the *phoP* mutant by the wild-type *phoP* gene restored all of the MICs to the wild type levels. Expression of PhoP was shown to be autoregulated and responsive to Mg^2+^ levels. The polymyxin B and gentamicin killing tests indicated that pretreatment of low Mg^2+^ can protect the wild-type *S*. *maltophilia* from killing but not *phoP* mutant. Interestingly, we found *phoP* mutant had a decrease in expression of SmeZ, an efflux transporter protein for aminoglycosides in *S*. *maltophilia*. Moreover, *phoP* mutant showed increased permeability in the cell membrane relative to the wild-type. In summary, we demonstrated the two-component regulator PhoP of *S*. *maltophilia* is involved in antimicrobial susceptibilities and low Mg^2+^ serves as a signal for triggering the pathway. Both the alteration in membrane permeability and downregulation of SmeZ efflux transporter in the *phoP* mutant contributed to the increased drug susceptibilities of *S*. *maltophilia*, in particular for aminoglycosides. This is the first report to describe the role of the Mg^2+^-sensing PhoP signaling pathway of *S*. *maltophilia* in regulation of the SmeZ efflux transporter and in antimicrobial susceptibilities. This study suggests PhoPQ TCS may serve as a target for development of antimicrobial agents against multidrug-resistant *S*. *maltophilia*.

## Introduction

*Stenotrophomonas maltophilia* is a nonfermentative gram-negative bacillus that may cause nosocomial infections, especially affecting immunocompromised patients who have been hospitalized for prolonged periods and received broad-spectrum antibiotic therapy [1–4]. Therapy of these infections presents a significant challenge because of the intrinsic resistance of *S*. *maltophilia* to most of the currently used antimicrobial agents including carbapenems [1–4]. Isolation of multidrug-resistant *S*. *maltophilia* in intensive care settings has also been noted with increasing frequency [1,2,4]. Several molecular mechanisms contribute to multi-drug resistance of *S*. *maltophilia*, including multidrug efflux pumps and plasmids or integrons carrying resistant determinants such as L1/L2 β-lactamases and aminoglycoside-modifying enzymes [1–3]. The resistance-nodulation-division (RND)-type efflux pump is a common cause of the multidrug resistance of *S*. *maltophilia*. Eight putative RND-type efflux systems (SmeABC, SmeDEF, SmeGH, SmeIJK, SmeMN, SmeOP, SmeVWX, and SmeYZ) were reported in the *S*. *maltophilia* [5]. In particular, it was noted that the SmIJK and SmeYZ pumps are constitutively expressed, and both are redundant in extrusion of aminoglycosides [6,7].

Cationic antimicrobial polypeptides (CAPs) are increasingly used to treat infections caused by multidrug-resistant bacteria. One of the important mechanisms of resistance to CAPs in gram-negative bacteria is modification of lipopolysaccharide (LPS) to remodel the composition of the outer membrane [8,9]. A polycistronic unit (*arnBCADTEF* or *pmrHFIJKLM*, called *arn* or *pmr* operon for short) is involved in LPS modification. Genes of the *arn* operon are necessary for biosynthesis and addition of 4-aminoarabinose (Ara4N) to the 4’ phosphate of lipid A- a modification contributing to a reduction in the net negative charge of LPS and consequently decreasing binding of CAPs to the outer membrane [8,9]. In a large number of gram-negative species, the genes involved in LPS modification, are regulated by the bacterial two-component systems (TCSs) [10–14]. The *Salmonella* PmrAB and PhoPQ TCSs are involved in resistance to the CAPs such as polymyxin B. Transcriptional activation of the *pmr* operon requires the PmrAB, PmrB the sensor kinase and PmrA the cognate response regulator [9]. The PmrAB of *Salmonella* is activated by Fe^3+^, which is sensed by the PmrB protein, and by low Mg^2+^, which is sensed by the PhoQ sensor protein. The low Mg^2+^ activation requires *pmrD*, a PhoPQ-regulated gene [13]. PhoP positively controls the *pmr* operon by increasing production of PmrD at the transcriptional level [13], which in turn activates the PmrA protein post-translationally, resulting in modification of LPS [12,13].

Seeing *S*. *maltophilia* polymyxin B resistance rate is up to 57.7% in the Asian-Pacific region [15] and MIC ranges of polymyxin B for clinical *S*. *maltophilia* isolates from National Taiwan University Hospital were 32–256 μg/ml (unpublished data), we investigated the role of PhoP in polymyxin B resistance. PhoP was shown to mediate polymyxin B resistance as expected. Surprisingly, we found PhoP is also associated with susceptibilities of *S*. *maltophilia* to other antimicrobials. Moreover, we found expression of SmeZ, an efflux transporter protein, was regulated by PhoP. This is the first report to describe the role of Mg^2+^-sensing PhoPQ TCS of *S*. *maltophilia* in regulation of the SmeZ efflux protein and in antimicrobial susceptibilities.

## Materials and Methods

### Bacterial strains, plasmids and growth condition

The bacterial strains and plasmids used in this study are listed in S1 Table. *S*. *maltophilia* S22 is a blood culture isolate from National Taiwan University Hospital. Bacteria were routinely cultured at 37°C in Luria-Bertani (LB) medium.

### Gene-knockout by homologous recombination

For construction of the *phoP* mutant, sequences flanking the *phoP* were amplified by PCR using primer pairs PhoPQout up971-F/PhoPQout up971-R and PhoPQout down1022-F/PhoPQout down1022-R (S2 Table), respectively and cloned into pGEM^®^-T Easy (Promega, USA) to generate pGphoP-up and pGphoP-dn. The pGphoP-up was digested with *Sal*I/*Xba*I and the *phoP* upstream sequence-containing fragment was ligated to the *Sal*I/*Xba*I-digested pGphoP-dn to produce the pGphoP-updn plasmid which contains both upstream and downstream sequences of *phoP*. The DNA fragment containing the combined upstream and downstream sequence of *phoP* was cleaved from pGphoP-updn by *Sal*I/*Sph*I and ligated into *Sal*I/*Sph*I-cleaved pEX18Tc [7] to generate pEX18phoP-updn. The plasmid (pEX18phoP-updn) was mobilized into *S*. *maltophilia* S22 via conjugation [7,16]. The deleted allele was transferred to the *S*. *maltophilia* chromosome by double-crossover homologous recombination through sequential selection of tetracycline (30 μg/ml) and then 10% sucrose. The resultant mutants with correct double-crossover events were verified by PCR and sequencing as described previously [7,16]. *smeZ* mutant was constructed in the same way using primer pairs SmeZout up-F/SmeZout up-R and SmeZout down-F/SmeZout down-R.

### Construction of the PhoP-complemented strain

Since *phoP* knockout lowered the expression of *phoQ* mRNA (data not shown), full length *phoPQ* was used to construct the PhoP-complemented strain. Full length *phoPQ* was amplified by PCR using primer pair PhoPQcomplementation-F and PhoPQcomplementation-R (S2 Table) and cloned into *Pst*I/*Kpn*I digested pRK415 [7] to generate the *phoP* complementation plasmid, pRKphoPQ. The pRKphoPQ was then transformed into the *phoP*-knockout mutant to generate the PhoP-complemented strain.

### MIC assay

In vitro determination of MICs for various drugs was performed by the broth microdilution method according to the guidelines proposed by Clinical and Laboratory Standard Institute [17].

### Reporter assay

The promoter region of *phoP* was amplified by the primer pair, phoPpromoterF/phoPpromoterR (S2 Table) and cloned into pGEM®-T Easy to generate pGphoPp. pGphoPp was cut with *Sac*I/*Pst*I and the promoter-containing fragment was ligated with the *xylE* containg pRK415 to construct the *phoP-xylE* reporter plasmid. The overnight cultures of the reporter plasmid-containing wild-type and *phoP* mutant were diluted to an OD_600_ of 0.1 in the same medium and the XylE activity was measured as described previously [18] at the time points indicated after incubation at 37°C.

### Purification of Glutathione S-transferase (GST)-tagged recombinant protein and electrophoretic mobility shift assay (EMSA)

The full-length *phoP* gene was cloned into plasmid pGEX-4T-1 by *Bam*HI and *Xho*I to generate plasmid pGEX-PhoP. Over-expression and purification of the GST-tagged PhoP was performed as described (GE Healthcare Life Sciences, USA). The purity of PhoP preparation was confirmed by SDS-PAGE. The promoter regions of *phoP* and *smeZ* were amplified by the primer pairs, phoPpromoterF/phoPpromoterR and *smeZ* EMSA-F/*smeZ* EMSA-R, respectively (S2 Table). The amplified promoter DNA fragments (0.1 μg each for the promoter DNA of *phoP* and *smeZ*) were incubated with 0, 0.25 or 0.5 μM GST-tagged PhoP protein in a 10-μl binding buffer [18]. After incubation for 30 min at room temperature, the reaction mixtures were loaded onto 5% non-denaturing polyacrylamide gels and the gel was electrophoresed at 100 V for 1.5–2 h before staining with ethidium bromide [18]. An EMSA using purified GST-tagged PhoP and the IRDye-labeled *phoP* promoter DNA was also performed. The unlabeled *phoP* promoter DNA fragments were included in the assay as the competitors to demonstrate the binding specificity of the *phoP* promoter DNA and GST-PhoP. The *phoP* promoter sequence was amplified from the pGphoPp using the IRDye® 700-labeled M13F/M13R primers (S2 Table, Integrated DNA Technologies). The DNA fragment amplified from the pGEM®-T Easy without the *phoP* promoter sequence by the same primer pair was used as the negative control. The amplified product (0.1 μg) was incubated with 0–0.5 μM GST-PhoP protein in a 10-μl binding buffer. After incubation for 30 min at room temperature, the reaction mixtures were analyzed as described above except that the gel image was obtained by the quantitative infrared fluorescent imaging system (Odyssey, LI-COR). For the competitive assay, the un-labeled *phoP* promoter DNA fragments (0.5, 1, 2 μg) amplified from pGphoPp using unlabeled M13 primers were included in the incubation solution.

### Real-time reverse transcription (RT)-PCR

To investigate whether Mg^2+^ is a signal of the PhoPQ TCS, we performed real-time RT-PCR to assess the expression of *phoP* using primers, phoP realtime-F and phoP realtime-R (S2 Table). Overnight cultures were diluted 100-fold, grown to OD_600_ 0.5 and supplemented with various concentrations of Mg^2+^ in N-minimal medium (5 mM KCl, 7.5 mM (NH_4_)_2_SO_4_, 0.5 mM K_2_SO_4_, 1 mM KH_2_PO_4_, 38 mM glycerol, 0.1% casamino acid, 0.1 M Tris-HCl, pH7.4). After incubation at 37°C for 5 h, total RNA was extracted and real-time RT-PCR was performed as described [18]. The mRNA levels were normalized against 16S rRNA. In addition, the effect of *phoP* mutation on expression of the nine efflux transporter genes (*smeB*, *smeE*, *smeH*, *smeJ*, *smeK*, *smeN*, *smeP*, *smeW*, and *smeZ*) was also examined in the same way without Mg^2+^ treatment using primers previously described [19].

### The polymyxin B and gentamicin killing tests for *S*. *maltophilia* in response to Mg^2+^ levels

We first tested the effect of pretreatment with various concentrations of Mg^2+^ on the survival of the wild-type after challenge with polymyxin B [18]. The overnight 3-ml LB culture was washed with N-minimal medium without Mg^2+^ three times and the pellet was resuspended in the same medium with Mg^2+^ at 0.01, 10 and 20 mM. After incubation at 37°C for 5 h, cells were washed twice, resuspended in 1-ml LB to OD_600_ 0.5 and incubated with polymyxin B (64 μg/ml) at 37°C for 1 h. The numbers of CFU were determined after serial dilution and plating on LB agar plates. The percent survival in polymyxin B was calculated as follows: (CFU of polymyxin B-challenged culture/CFU of no challenge culture) x 100%.

To further test the effects of pretreatment with Mg^2+^ on the survival of the wild-type, *phoP* mutant and *phoP*-complemented strain in drugs, we performed the polymyxin B and gentamicin killing tests after pretreatment with low (10 μM) and high (10 mM) concentrations of Mg^2+^. In the polymyxin B killing test, bacterial cells were incubated for 1 h in the presence or absence of polymyxin B (64 and 128 μg/ml) after pretreatment with Mg^2+^, while the wild-type, *phoP* mutant and phoPc strain were challenged or not with Gm at concentrations of 0.5x and 1x MICs for each strains after the same pretreatment in the gentamicin killing test. The relative survival (low Mg^2+^ to high Mg^2+^) was from the percent survival in low Mg^2+^divided by the percent survival in high Mg^2+^. The percent survival was calculated as described above.

### ANS (8-anilino-1-naphthylenesulfonic acid) membrane permeability assay [20]

To assess the integrity of bacterial cell membranes, the fluorescent probe, 8-anilino-1-naphthylenesulfonic acid (Sigma-Aldrich, USA) was used. ANS is a hydrophobic probe that exhibits enhanced fluorescence in nonpolar/hydrophobic environments. Overnight cultures were diluted 100-fold in 5 ml fresh LB medium and grown to an OD_600_ of 0.5 at 37°C. A 1-ml cell culture was harvested by centrifugation and washed with phosphate buffered saline (PBS). Cells were then resuspended in 1-ml PBS containing ANS (final concentration 3 μM). The solution was kept at room temperature for 10 min in the dark and the fluorescence intensity of cells was quantified using a microplate reader with 375 nm excitation and 510 nm emission.

### Triton X-100 sensitivity assay [21]

Overnight cultures were diluted 100-fold in fresh LB medium and grown to an OD_600_ of around 0.2 at 37°C. Triton X-100 (0.5%) was added or not into the bacterial culture and the growth was monitored by OD_600_ determination at 30-min intervals after further incubation at 37°C.

### Detection of aminoglycoside-modifying enzymes by the agar diffusion method

We detected the presence of aminoglycoside-modifying enzymes in wild-type *S*. *maltophilia* and *phoP* mutant using a protocol modified by Gad et al. [22]. Briefly, crude enzyme extracts were prepared after sonication of bacterial suspensions and centrifugation to obtain the supernatant. For a total of 50 μl reaction mixture, 25 μl of the enzyme extract (containing 1 mg/ml protein) or phosphate buffer saline (PBS, as the control) was incubated for 3 h in the presence of 22 nmoles of the tested aminoglycoside antibiotics, 50 nmoles dithiothreitol, 2.5 μmoles Tris pH 8.1, 120 nmoles ATP and 0.4 μmoles MgCl_2_. To monitor the extent of inactivation of the antibiotics, the reaction mixture was spotted on filter paper disks (10 μl per disk), which were placed on Mueller-Hinton agar plates seeded with a sensitive *E*. *coli* DH5α strain. Plates were incubated at 37°C for 24 h. In case of inactivation of the antibiotics, the presence of a crude enzyme extract reduces the inhibition zone (diameter) of the disk relative to that of the control disk (PBS). For method validation, two clinical isolates of *E*. *coli* (resistant to gentamicin and amikacin, respectively) and *E*. *coli* DH5α carrying a kanamycin resistance plasmid were included in the assay.

### Nucleotide sequence accession numbers

The *phoP* and *phoQ* nucleotide sequences of *S*. *maltophilia* S22 have been deposited in GenBank under accession no. KT001239.

## Results

### Identification of PhoP and PhoQ in *S*. *maltophilia*

Many TCSs, including PhoPQ and RppAB, are known to regulate tolerance to CAPs in pathogenic bacteria [12,23,24]. Knowing MICs of polymyxin B, a naturally occurring CAP, may range from 32–256 μg/ml in clinical isolates of *S*. *maltophilia* (unpublished data), we investigated whether *S*. *maltophilia* PhoP protein is involved in tolerance to polymyxin B. First, we searched for *phoP* and *phoQ* homologues in the released *S*. *maltophilia* K279a genome and found the *phoP* and *phoQ* counterparts. We then amplified *phoPQ* genes using the genomic DNA of a clinical polymyxin B-resistant isolate (S22, MIC 128 μg/ml) with primers designed from the *S*. *maltophilia* K279a genome sequence and analyzed the product sequences. S1 Fig shows the alignment of the *S*. *maltophilia* S22 PhoP and PhoQ proteins with those of other bacteria. *S*. *maltophilia* S22 PhoP and PhoQ proteins share sequence identity with their counterparts in *S*. *maltophilia* K279a (92% and 99%), *Xanthomonas campestris* NCPPB 2251 (91% and 77%), *Xanthomonas oryzae* pv. oryzae KACC 10331 (91% and 76%), *Pseudomonas aeruginosa* PAO1 (55% and 34%) and *Salmonella enterica* serovar Typhimurium LT2 (41% and 33%).

### Increased susceptibilities of *S*. *maltophilia phoP* mutant to polymyxin B and other antimicrobial agents

To demonstrate the role of *phoP* gene in regulating polymyxin B susceptibility, we constructed *phoP* mutant through allelic exchange mutagenesis (see Materials and Methods). Growth was not affected in the *phoP* mutant (S2 Fig). We found *phoP* mutant was 64-fold more susceptible to polymyxin B than the wild-type *S*. *maltophilia* S22 (MIC 2 vs 128 μg/ml) (Table 1). MICs of other drugs (except amikacin) tested for *phoP* mutant also decreased 2–16 fold (Table 1) relative to the wild-type, with an 8- and a 16-fold decrease in MIC for kanamycin and gentamicin, respectively. The complemented strain had restored MIC levels as the wild-type (Table 1).

**Table 1:** MICs of antimicrobial agents against wild-type *S*. *maltophilia* S22 and its derivatives. wt, wild-type *S*. *maltophilia* S22; phoP, *phoP* mutant; phoPc, PhoP-complemented strain; smeZ, *smeZ* mutant; PB, polymyxin B; Gm, gentamicin; Km, kanamycin; Sm, streptomycin; Amk, amikacin; Spec, spectinomycin; Amp, ampicillin; Cm, chloramphenicol.

	PB	Gm	Km	Sm	Amk	Spec	Amp	Cm
MIC (μg/ml)								
wt	128	64	256	1024	256	4096	4096	16
phoP	2	4	32	256	256	2048	2048	4
phoPc	128	64	256	1024	256	4096	4096	16
smeZ	64	16	128	256	64	4096	4096	16

### Autoregulation and the response to Mg^2+^ levels of *S*. *maltophilia* PhoP

**Fig 1:**
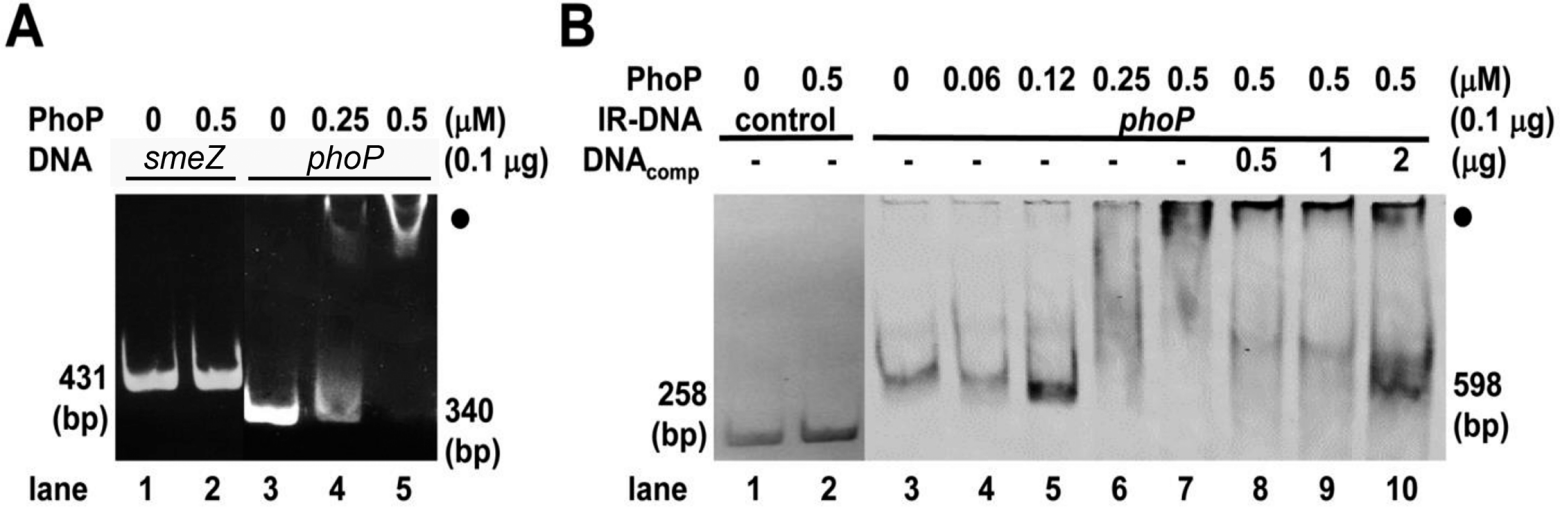
The binding site of *S*. *maltophilia* PhoP revealed by electrophoretic mobility shift assay. **(A) The purified GST-PhoP with the *smeZ* or *phoP* promoter DNA fragment.** DNA fragments (0.1 μg) of the *smeZ* promoter (431 bp) or the *phoP* promoter (340 bp) obtained by PCR were incubated with the indicated concentrations (0, 0.25 or 0.5 μM) of PhoP protein. After protein-DNA complex formation, the fragments were resolved on a 5% non-denaturing polyacrylamide gel. Lane 1, *smeZ* promoter DNA; lane 2, *smeZ* promoter with PhoP; lane 3, *phoP* promoter DNA; lanes 4–5, *phoP* promoter with PhoP. **(B) The purified GST-PhoP with the IRDye-labeled *phoP* promoter DNA fragment.** IRDye-labeled DNA fragments (IR-DNA, 0.1 μg) of the *phoP* promoter (598 bp) or the negative-control (258 bp) were incubated with the indicated concentrations (0–0.5 μM) of PhoP protein. The fragments were analyzed as described in Materials and Methods. Lane 1, IRDye-labeled negative control DNA; lane 2, IRDye-labeled negative control DNA with PhoP; lane 3, IRDye-labeled *phoP* promoter DNA; lanes 4–7, IRDye-labeled *phoP* promoter DNA with PhoP; lanes 8–10, IRDye-labeled *phoP* promoter DNA with PhoP and un-labeled *phoP* promoter DNA (competitive DNA, DNA_comp_). Closed circle in A and B, the protein-promoter DNA complex.

**Fig 2:**
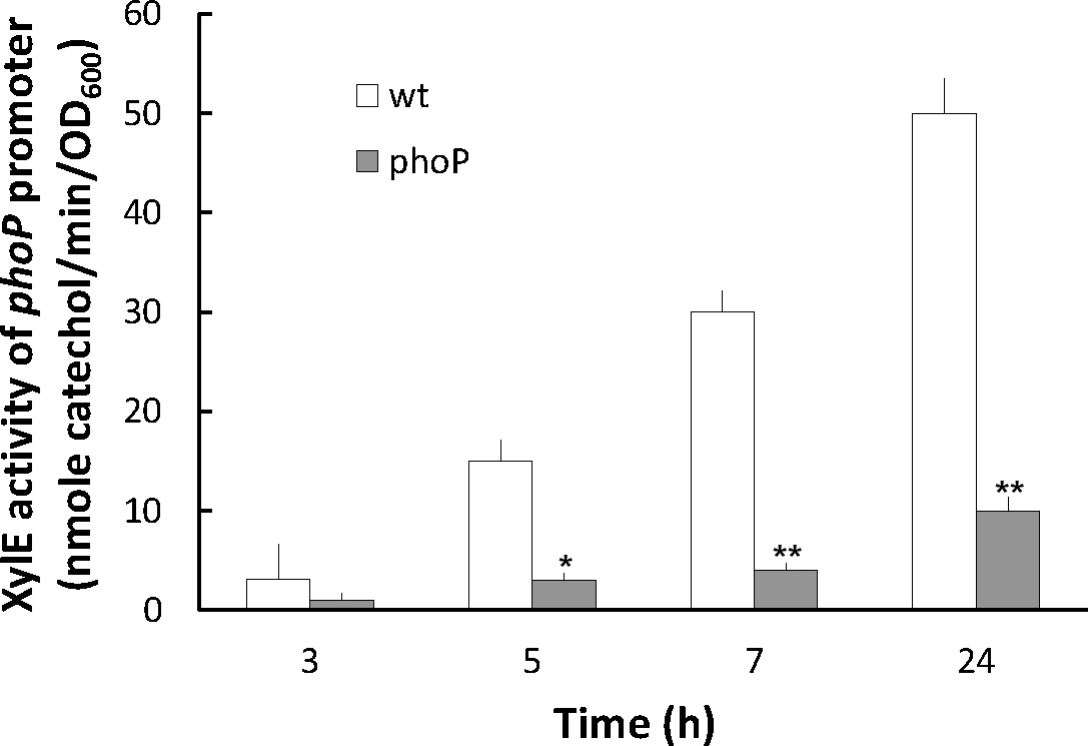
The promoter activity of *phoP* in wild-type *S*. *maltophilia* and *phoP* mutant. The activities of XylE in the *phoP*-*xylE* reporter plasmid-transformed wild-type and *phoP* mutant were determined by the reporter assay after incubation for 3, 5, 7 and 24 h. The data are the averages and standard deviations of three independent experiments. Significant difference between wild-type and *phoP* mutant was observed by Student’s *t*-test analysis (*, P<0.01; **, P<0.001). wt, wild-type; phoP, *phoP* mutant.

*Salmonella* PhoPQ [12] and *Proteus* RppAB [24], both involved in PB susceptibility, have been shown to sense Mg^2+^ levels and to be autoregulated. To study whether PhoP is autoregulated by binding to the *S*. *maltophilia phoP* putative promoter, an EMSA was performed. The data in Fig 1A indicated that the purified GST-tagged PhoP proteins could bind specifically to the *phoP* promoter DNA fragment but not to the *smeZ* promoter DNA fragment (compare lanes 1–2 to lanes 3–5). Another EMSA with purified GST-tagged PhoP and the IRDye-labeled *phoP* promoter DNA fragment showed that a band shift was visible when the labeled *phoP* promoter DNA was incubated with enough GST-PhoP protein (lanes 3–5 vs 7 in Fig 1B) and the unlabeled *phoP* promoter DNA can act as a competitor to reduce the binding of the IRDye-labeled *phoP* promoter DNA with GST-PhoP (lane 7 vs 8–10 in Fig 1B). No shift band was observed when the IRDye-labeled negative control DNA was incubated with GST-PhoP (lanes 1–2 in Fig 1B). In addition, the wild-type carrying the *phoP*-*xylE* reporter plasmid also displayed higher XylE activity compared to the *phoP* mutant carrying the same reporter plasmid (Fig 2). Therefore, expression of *S*. *maltophilia phoP* gene is likely autoregulated by itself.

**Fig 3:**
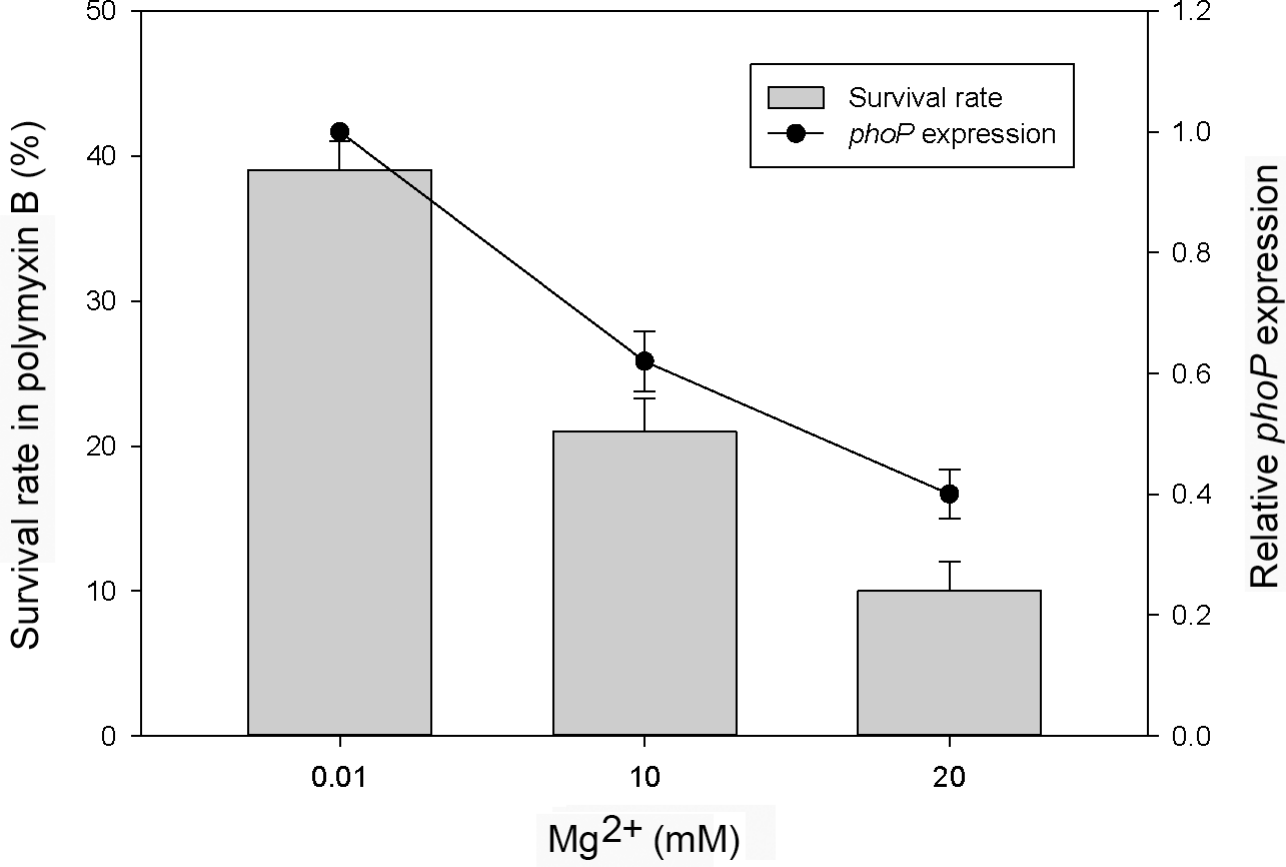
Survival in polymyxin B and *phoP* expression of wild-type *S*. *maltophilia* S22 in response to Mg^2+^. Left axis shows the survival rate of wild-type *S*. *maltophilia* in the presence of polymyxin B (PB, 64 μg/ml) for 1 h after pretreatment with Mg^2+^ at 0.01, 10 and 20 mM for 5 h. Right axis shows the relative *phoP* expression of the wild-type after the same pretreatment. The *phoP* expression in pretreatment with Mg^2+^ at 0.01 mM was set at 1. The percent survival was calculated using the following formula: (CFU of PB-challenged culture/CFU of no-challenge culture) x 100%. The data represent the averages and standard deviations of three independent experiments.

Knowing PhoP is likely autoregulated, we then investigated the expression of *phoP* in the wild-type *S*. *maltophilia* in the presence of different concentrations of Mg^2+^ (in N-minimal medium) by the real-time RT-PCR assay. The *phoP* mRNA level of *S*. *maltophilia* was reduced by high Mg^2+^ at 20 mM to around 40% of the level in low Mg^2+^ at 0.01 mM (right axis in Fig 3). Thus, low Mg^2+^ may serve as a signal to activate the expression of *S*. *maltophilia* PhoP.

### Low Mg^2+^ pretreatment protecting the wild-type *S*. *maltophilia* but not *phoP* mutant from killing by polymyxin B or gentamicin

**Fig 4:**
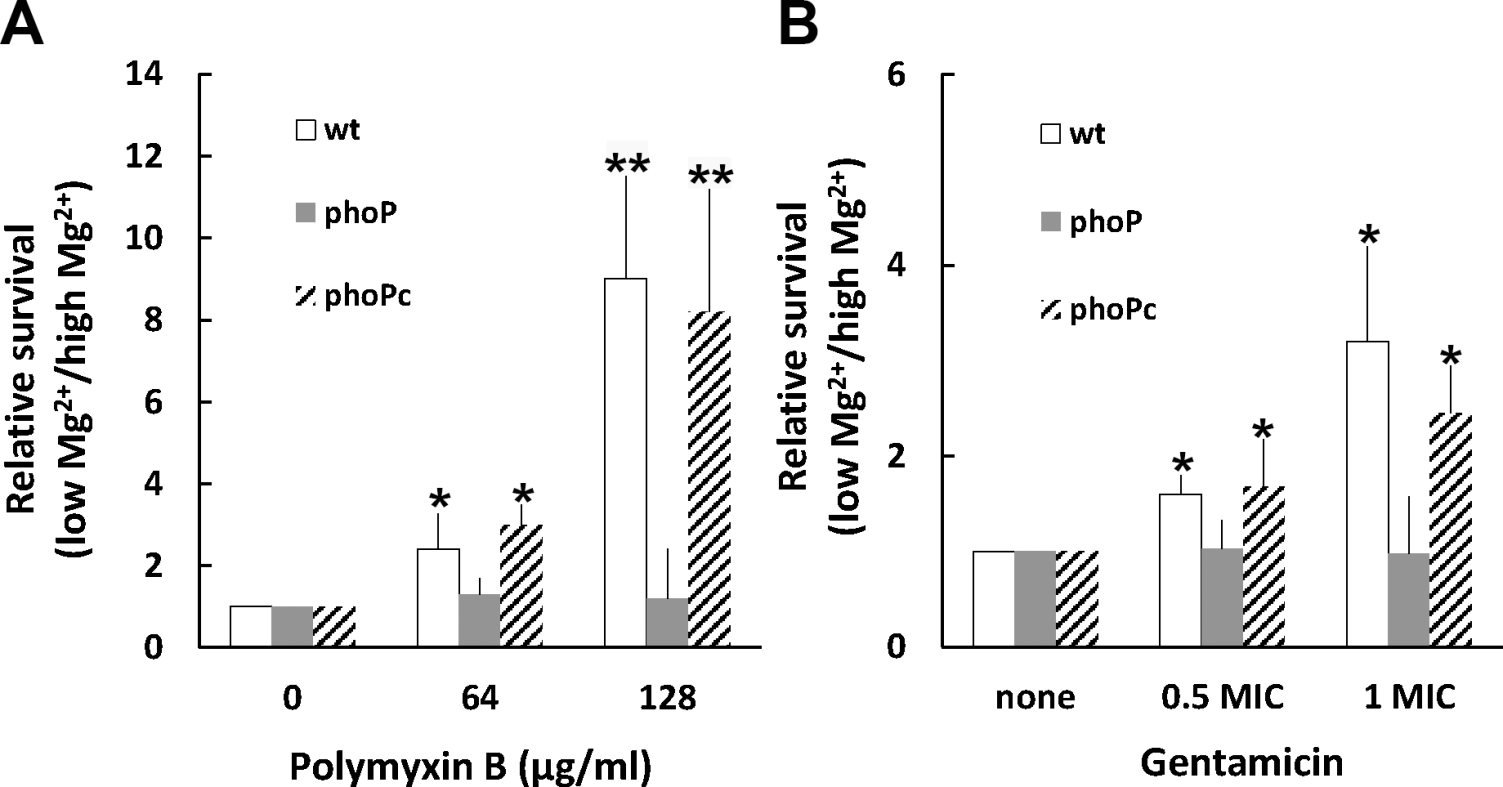
**Relative survival of *S*. *maltophilia* in polymyxin B (PB) (A) or gentamicin (Gm) (B) after pretreatment with low and high Mg**^**2+**^. We performed the PB and Gm killing tests for the wild-type *S*. *maltophilia* (wt), *phoP* mutant (phoP) and *phoP*-complemented strain (phoPc) after pretreatment with low (10 μM) and high (10 mM) concentrations of Mg^2+^. In the PB killing assay, bacterial cells were challenged with PB (64 and 128 μg/ml) or not. In the gentamicin killing test, bacterial cells were challenged or not with Gm at concentrations of 0.5x and 1x MICs for each strains. The relative survival was calculated as described in Materials and Methods. The relative survival for the wild-type, *phoP* mutant and phoPc strain without challenge with PB or Gm was set at 1. The data represent the averages and standard deviations of three independent experiments. Significant difference between the relative survival of the drug challenge and no challenge for the wild-type and the phoPc strain was observed by Student’s t-test analysis (*, p<0.05; **, p<0.005).

Knowing *S*. *maltophilia* PhoP regulates drug susceptibility and PhoP expression is responsive to Mg^2+^, we performed the MIC assay of polymyxin B and gentamicin after overnight pretreatment of *S*. *maltophilia* with low (10 μM) and high Mg^2+^ (10 mM) in N-minimal medium. Compared to high Mg^2+^, low Mg^2+^ pretreatment resulted in an increase in MICs of both drugs for the wild-type but not the *phoP* mutant (Table 2). Moreover, we found polymyxin B MIC for the wild-type increased 2-fold in the presence of the PhoP-expressing plasmid. To further demonstrate if PhoP induced by low Mg^2+^ can protect *S*. *maltophilia* from antibiotic killing, the polymyxin B killing assay was performed after pretreating wild-type cells with Mg^2+^ at 0.01, 10, and 20 mM. The survival rate in polymyxin B increased about 4-fold after pretreating with Mg^2+^at 0.01 mM relative to that of pretreating with Mg^2+^at 20 mM (left axis in Fig 3). Moreover, we tested the effect of pretreatment of low Mg^2+^ on the survival of wild-type *S*. *maltophilia*, *phoP* mutant and *phoP*-complemented strain (phoPc) in either polymyxin B or gentamicin. The percent survival of *phoP* mutant pretreated with low and high Mg^2+^ for 5 h was almost the same (i.e. relative survival rate of low to high Mg^2+^ pretreatment near 1) after exposure to polymyxin B (64 or 128 μg/ml), while the wild-type and phoPc strains pretreated in the same way exhibited an increase in survival in low Mg^2+^, particularly in polymyxin B of 128 μg/ml (Fig 4A). In the gentamicin killing assay, the same pretreated-bacterial cells were challenged or not with Gm at concentrations of 0.5x and 1x MICs for each strains and the relative survival rate (low to high Mg^2+^) were determined. Again, the relative survival rate in gentamicin for *phoP* mutant was near 1 and more than 1 for the wild-type and the phoPc strain (Fig 4B). Together, these data indicate low Mg^2+^ may protect *S*. *maltophilia* from killing by polymyxin B and gentamicin through a PhoP-dependent pathway.

**Table 2:** MICs of polymyxin B and gentamicin for wild-type *S*. *maltophilia* S22 and *phoP* mutant pretreated with high and low Mg^2+^. high Mg, 10 mM Mg^2+^; low Mg, 10 μM Mg^2+^; wt, phoP, PB and Gm, the same as in Table 1.

	wt		phoP	
	high Mg	low Mg	high Mg	low Mg
MIC (μg/ml)				
PB	32	256	2	2
Gm	32	128	4	4

### Regulation of *S*. *maltophilia smeZ*, a gene encoding an efflux pump protein, by PhoP

**Fig 5:**
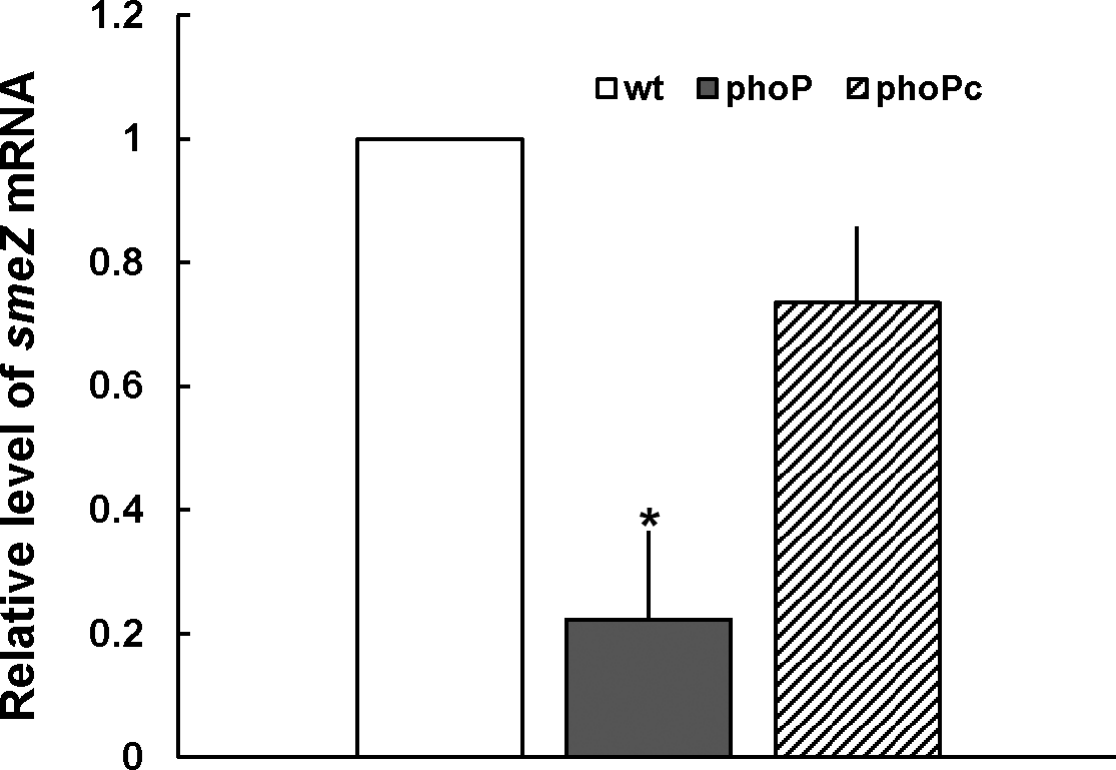
The effect of *phoP* knockout on the levels of *smeZ* mRNA in *S*. *maltophilia*. The *smeZ* mRNA amounts in the wild-type (wt), *phoP*-knockout mutant (phoP), and *phoP*-complemented strains (phoPc) were quantified by real-time RT-PCR. The value obtained for the wild-type cells was set at 1. The data represent the averages and standard deviations of three independent experiments. Significant difference between the levels of wild-type and *phoP* mutant was observed by Student’s *t*-test analysis (*, p<0.05).

It has been reported that bacteria can use the TCS in disposing chemicals, including antibiotics, through regulating the efflux pumps [11,25–28]. Therefore, we investigated if *S*. *maltophilia* PhoP would regulate efflux pumps to affect antimicrobial susceptibilities. Inner membrane transporter genes (*smeB*, *smeE*, *smeH*, *smeJ*, *smeK*, *smeN*, *smeP*, *smeW*, and *smeZ*) of each RND efflux system were selected as PhoP-regulated targets for real-time RT-PCR to evaluate the expression of each pump in the wild-type, *phoP* mutant and complemented strain (phoPc). The results revealed only *smeZ* expression was significantly low in the *phoP* mutant relative to the wild-type and phoPc strain (Fig 5). Since SmeZ has been known to participate in extrusion of aminoglycosides [7], we examined aminoglycoside MICs for *S*. *maltophilia* S22 *smeZ* mutant. As expected, aminoglycoside (except spectinomycin) MICs for *smeZ* mutant were 2–4 fold lower than those of the wild-type. Loss of *smeZ* didn’t affect MICs of ampicillin and chloramphenicol but did alter polymyxin B MIC (Table 1). Together, the data suggest that PhoP could affect aminoglycoside susceptibilities through regulating expression of SmeZ transporter. We observed MICs of gentamicin, kanamycin and streptomycin decreased both for *phoP* mutant and *smeZ* mutant relative to the wild-type (Table 1). It is worth noting that except amikacin and streptomycin, all drugs tested showed increased potency for the *phoP* mutant relative to the *smeZ* mutant (Table 1).

### Increased membrane permeability in *phoP* mutant

**Fig 6:**
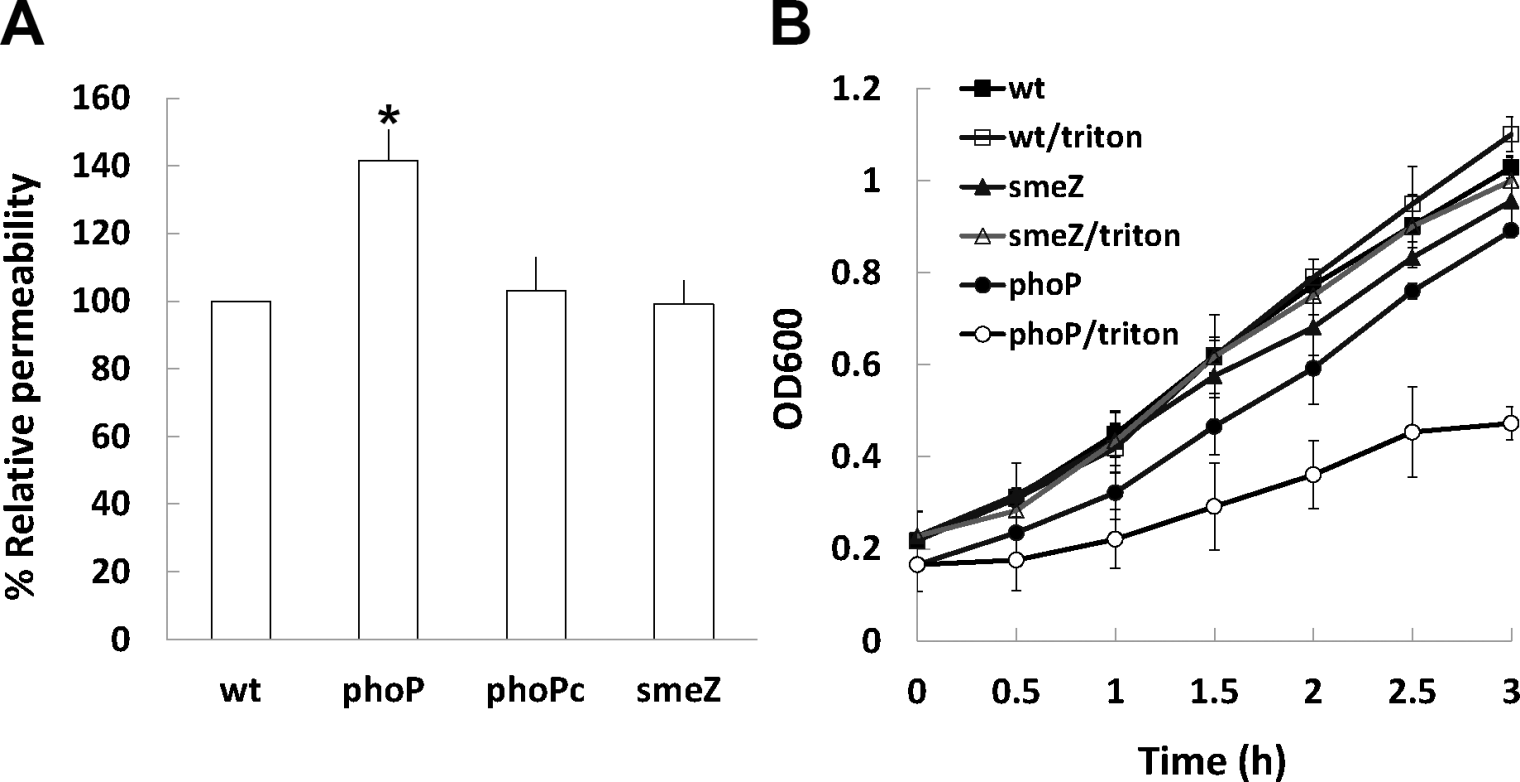
**(A) The membrane permeability of the wild-type *S*. *maltophilia* (wt), *phoP* mutant (phoP), *phoP*-complemented strain (phoPc) and *smeZ* mutant (smeZ) by the ANS assay.** Overnight cultures were grown, washed and resuspended in the ANS solution (final concentration 3 μM). The fluorescence intensity of cells was quantified using a microplate reader. The data represent the averages and standard deviations of three independent experiments. Significant difference between the percent relative permeability of wild-type and *phoP* mutant was observed by Student’s *t*-test analysis (*, p<0.05). **(B) The effect of Triton X-100 on the growth of the wild-type *S*. *maltophilia*, *phoP* mutant and *smeZ* mutant.** Overnight cultures were grown and treated with 0.5% triton X-100 or not. The growth was then monitored by measuring OD_600_ at 30-min intervals. The data represent the averages and standard deviations of three independent experiments.

PhoPQ-mediated regulation was shown to produce a more robust permeability barrier in the outer membrane of *S*. *enterica* Serovar Typhimurium and *Neisseria* [14,29]. They found that *phoP* null mutant produces an outer membrane severely compromised in its barrier function that is important in making the pathogen more resistant to the stresses, including antimicrobial agents. In view of the increased drug susceptibilities of *S*. *maltophilia phoP* mutant, the fluorescent probe, 8-anilino-1- naphthylenesulfonic acid (ANS), was used to assess the integrity of the bacterial outer membrane. We found the outer membrane permeability of *phoP* mutant increased compared to that of the wild-type, phoPc strain and *smeZ* mutant (Fig 6A). It seems that somehow the barrier function of the outer membrane in the mutant is disturbed. To further characterize the change in permeability, Triton X-100, a nonionic surfactant, was used to test its effect on the wild-type, *phoP* mutant and phoPc strain. Similarly, we found the *phoP* mutant was more sensitive to the treatment of Triton X-100 than the wild-type and *smeZ* mutant (Fig 6B). Together, the data indicate the permeability change may be also one of the reasons for the susceptibilities of *phoP* mutant to the drugs tested. Comparison of aminoglycoside MICs for the *phoP* mutant and the *smeZ* mutant (Table 1), it is likely that both the cell permeability barrier and SmeZ extrusion mediated by PhoP contribute to aminoglycoside resistance in *S*. *maltophilia*.

### The increase of aminoglycoside susceptibility in *phoP* mutant was not related to the absence of aminoglycoside-modifying enzymes

Because aminoglycoside-modifying enzyme is an important consideration for aminoglycoside resistance in *S*. *maltophilia*, we assessed if aminoglycoside- modifying enzymes exist in *phoP* mutant by the agar diffusion method. Table 3 shows that the extent of inactivation of gentamicin, amikacin and kanamycin by enzyme extracts from wild-type *S*. *maltophilia* and *phoP* mutant was similar to that of PBS control. In contrast, the extracts from a gentamicin and an amikacin resistant clinical isolates of *E*. *coli* exhibited a significant inactivation of gentamicin and amikacin, respectively (1.98 vs 2.47 cm and 2.13 vs 2.62 cm). In addition, the extract from *E*. *coli* DH5α carrying a kanamycin resistance plasmid showed a significant inactivation of kanamycin compared to the PBS control (1.58 vs 2.13 cm). The results indicate the absence of aminoglycoside-modifying enzymes was not the reason for the increase in aminoglycoside susceptibility of *phoP* mutant.

**Table 3:** The extent of inactivation of gentamicin, amikacin and kanamycin by enzyme extracts from wild-type *S*. *maltophilia*, *phoP* mutant and *E*. *coli*. wt, phoP, Gm, Amk and Km, the same as in Table 1; *, a gentamicin (E1) and an amikacin (E2) resistant clinical isolate of *E*. *coli*; E3, *E*. *coli* DH5α carrying a kanamycin resistance plasmid; PBS, phosphate buffer saline control. The data are the averages of three independent experiments. ^#^, significant difference from the PBS control was observed by Student’s *t*-test analysis (P<0.01).

	Inhibition zone (cm)					
	wt	phoP	*E1	*E2	E3	PBS
Gm	2.45	2.47	1.98^#^	-	-	2.47
Amk	2.66	2.7	-	2.13^#^	-	2.62
Km	2.15	2.14	-	-	1.58^#^	2.13

## Discussion

The PhoPQ TCS governs the adaptation to low Mg^2+^ environments and other stress conditions by regulating expression of as much as 1% of the genes in many gram- negative bacterial species [11,12,30]. For example, CAP resistance is known to be regulated by the PhoPQ system in response to Mg^2+^ [10–13]. In this study, we described that PhoP could regulate antimicrobial susceptibilities in *S*. *maltophilia*. On one hand, the PhoP-mediated SmeZ expression could extrude aminoglycosides; on the other the cell permeability barrier regulated by PhoP also may contribute to drug susceptibilities. Several lines of evidence suggest that *S*. *maltophilia* PhoP should be the PhoP counterpart in other bacteria. First, sequence analysis of *S*. *maltophilia* PhoP realveals homology with other PhoP proteins, up to 91% identity and 92% similarity to *Xanthomonas oryzae* PhoP which has been shown to confer tolerance to CAPs in response to Mg^2+^ [23]. Second, *S*. *maltophilia phoP* mutant exhibited increased sensitivity to PB. Third, *S*. *maltophilia* PhoP expression is autoregulated and can be activated by low Mg^2+^.

It is becoming increasingly clear that the TCS-mediated stress responses are linked to antimicrobial resistance [31]. In this regard, a number of TCSs of important bacterial pathogens, such as CesRK of *Listeria monocytogenes* [32], CpxAR of *Klebsiella pneumoniae* [28], AdeRS and BaeSR of *Acinetobacter baumannii* [25,26], and PprBA and PhoPQ of *P*. *aeruginosa* [11,33] were shown to regulate antibiotic susceptibility. Seeing PhoPQ mediates responses to stresses, such as growth-limiting Mg^2+^, acids and antimicrobial peptides [12,23], it seems reasonable for us to discover *S*. *maltophilia* PhoP is involved in drug susceptibility.

How could a TCS affect drug tolerance? First, several efflux pumps have been shown to be regulated by the TCS [11,25–28]. In *E*. *coli*, CpxR-induced MarA activates expression of the AcrAB/TolC tripartite multidrug pump, thus leading to drug resistance [34]. For the first time, we found PhoP regulates expression of the efflux transporter SmeZ (Fig 5). Although the EMSA strongly suggested the lack of PhoP interaction with *smeZ* promoter region of *S*. *maltophilia* (Fig 1A), the result may be due to low affinity between them or a co-factor needed. Second, the TCS-mediated surface changes may alter cell permeability and thus affect drug susceptibility [29,33,35,36]. In view of PhoPQ-mediated regulation producing a more robust permeability barrier in the outer membrane of *S*. *enterica* serovar Typhimurium [29], the finding of increased membrane permeability in *S*. *maltophilia phoP* mutant (Fig 6) may explain the increased susceptibility of the mutant to the drugs tested in this study. The increased permeability of the outer membrane (by ANS test) may be indicative of a general decrease in outer membrane integrity in the *S*. *maltophilia phoP* mutant and thus a greater susceptibility to membrane perturbation by Triton X-100, lending support to the view of a membrane stabilization role for *S*. *maltophilia* PhoP. Cell membrane permeability-associated drug susceptibilities may arise from changes in outer membrane proteins to limit the drug access to bacterial cells [33,36,37] and TCSs may influence the expression and/or membrane localization of a number of membrane proteins to regulate bacterial cell permeability [33]. Nevertheless, our preliminary data indicated no obvious difference in the profile of outer membrane proteins between wild-type and *S*. *maltophilia phoP* mutant (data not shown). In addition, PhoPQ-mediated LPS modification is known to be associated with outer membrane permeability and CAP susceptibility [35]. More efficient permeability barrier in the PhoP-constitutive mutant has also been confirmed by higher drug resistance and the known PhoP-dependent lipid A modifications explain most of the alterations in the outer membrane permeability [29]. In this regard, analysis of the LPS profile of *S*. *maltophilia phoP* mutant is ongoing.

One susceptibility profile in Table 1 is affected by *smeZ* mutation only (amikacin) and the other by both *phoP* and *smeZ* mutation (streptomycin, gentamicin, kanamycin, polymyxin B). Among the latter, streptomycin tolerance is possibly through PhoP-mediated SmeZ extrusion only (the same MICs for *phoP* and *smeZ* mutants). However, PhoP-mediated both SmeZ-dependent and -independent pathways may confer tolerance to gentamicin and kanamycin (MIC for *phoP* mutant lower than *smeZ* mutant). In view of the antibacterial mechanism of PB and constitutive expression of *S*. *maltophilia* SmeZ [7], increased PB susceptibility in *smeZ* mutant should be due to the membrane disturbance [38]. As expected, *S*. *maltophilia smeZ* mutant (defective in aminoglycoside extrusion) has increased susceptibilities to streptomycin, gentamicin, kanamycin and amikacin. The limited contribution of SmeZ to kanamycin and amikacin susceptibilities is similar to the report of Crossman et al. [5] but much smaller than that reported by Lin et al. [7]. Interestingly, we found loss of *S*. *maltophilia phoP* decreased MICs of all aminoglycosides tested except amikacin. Based on the PhoP-mediated SmeZ expression shown in Fig 5, it doesn’t make sense to see amikacin susceptibility is affected by *smeZ* mutaion but not *phoP* mutation. In this regard, QseEF and QseBC TCSs were shown to be able to sense the same signal (epinephrine) to regulate formation of attaching and effacing lesion in Enterohemorrhagic *E*. *coli* [39]. Hence it is feasible that amikacin may induce other systems to compensate for the defect of SmeZ in the *S*. *maltophilia phoP* mutant. Regarding to this issue, a TCS operon is located upstream of *smeZ*, work is under way to disclose the role of the system in SmeZ-mediated drug resistance.

Several lines of evidence support that the Mg^2+^-sensing PhoPQ system can regulate expression of the LPS-modification operon, *arn*, leading to polymyxin B resistance [11,12]. Both *pmrD* gene and *arn* operon are not present in the released *S*. *maltophilia* genome. In *S*. *maltophilia* S22, we did find homologues of *ugd* and *galU* which have been shown to be involved in LPS modification and polymyxin B resistance [40] but both are not regulated by PhoP (data not shown). Systematic approaches to dissect the PhoP regulon relating to susceptibility to polymyxin B are under way.

In conclusion, for the first time, we reported *S*. *maltophilia* PhoP was involved in drug susceptibilities. It is likely that *S*. *maltophilia* PhoP regulates expression of downstream genes which may affect membrane permeability and expression of SmeZ efflux protein in response to a certain environmental cue, low Mg^2+^, thus resulting in drug tolerance. Further studies aimed at defining genes that fall into the PhoP regulon, as well as unravelling their complex interaction, should greatly aid our understanding of antibiotic resistance in *S*. *maltophilia*. This study highlights the possibility to develop antimicrobial agents against multidrug-resistant *S*. *maltophilia* by targeting the PhoPQ TCS.

## Supporting Information

S1 Fig **Alignment of the PhoP (A) and PhoQ (B) proteins of *S*. *maltophilia* with other response regulators and sensor kinases.** *Pseudomonas aeruginosa*, *Xanthomonas campestris*, *Xanthomonas oryzae*, *Escherichia coli* and *Salmonella* Typhimurium were included in alignment using the NASTAR-MegAlign program.
(TIF)

S2 Fig Effect of *phoP* deletion on the growth of *S*. *maltophilia*. The bacterial growth was expressed as the optical density at 600 nm (OD600). Overnight bacterial cultures of wild-type (wt) and *phoP* mutant (phoP) were diluted and regrown to the optical density of around 0.2 (OD600) and the growth was monitored at 1-h intervals. The data represent the averages and standard deviations of three independent experiments.
(TIF)

S1 Table Bacterial strains and plasmids used in this study. (DOCX)

S2 Table Primers used in this study. (DOCX)
