# Supplementary figures and images for: Stenotrophomonas maltophilia PhoP, a Two-Component Response Regulator, Involved in Antimicrobial Susceptibilities
Source: PLoS One. 2016 May 9;11(5):e0153753. doi: 10.1371/journal.pone.0153753 (PMC4861329; doi:10.1371/journal.pone.0153753)

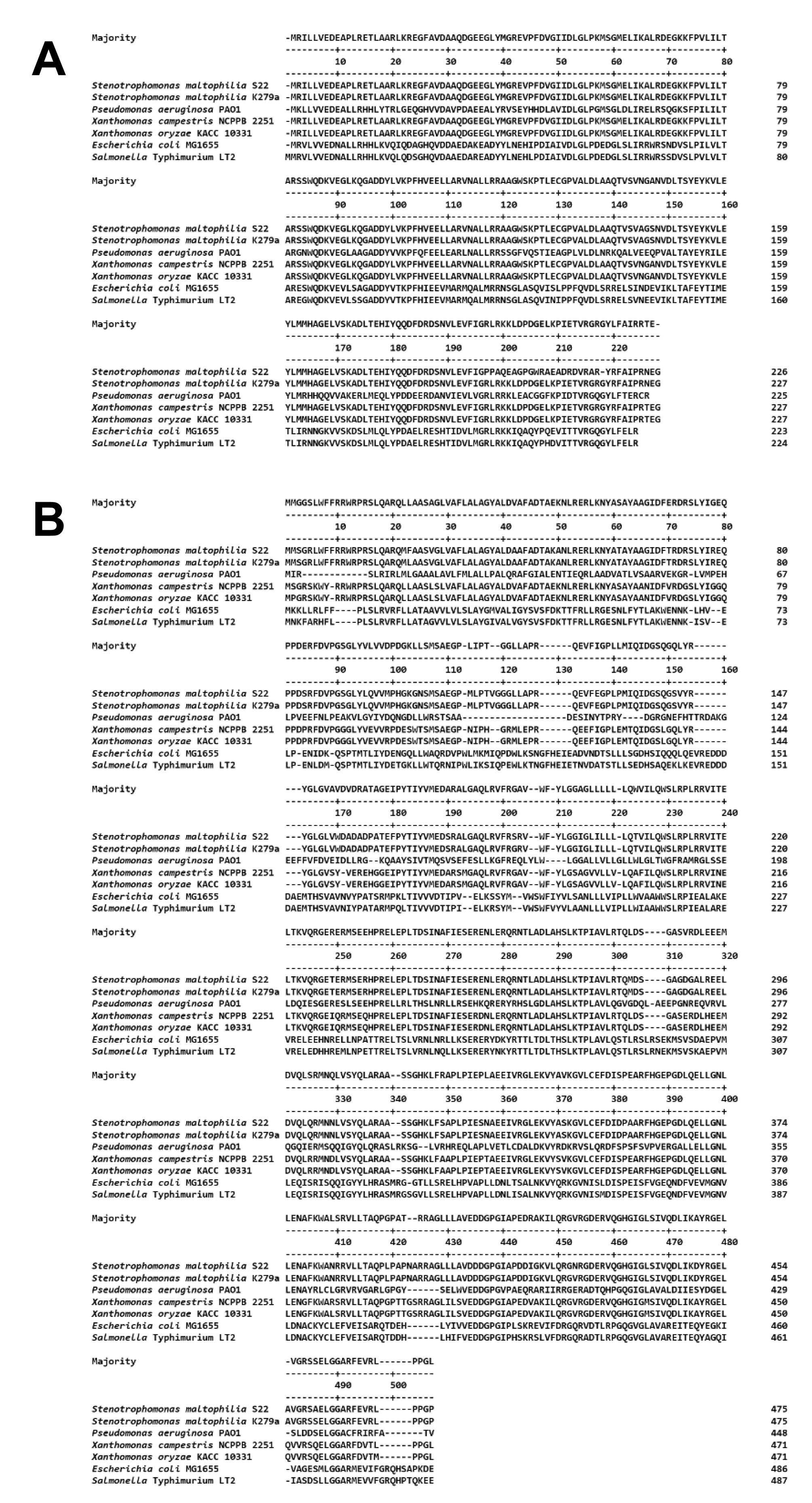

Supplement: S1 Fig — Alignment of the PhoP (A) and PhoQ (B) proteins of S. maltophilia with other response regulators and sensor kinases. Pseudomonas aeruginosa, Xanthomonas campestris, Xanthomonas oryzae, Escherichia coli and Salmonella Typhimurium were included in alignment using the NASTAR-MegAlign program. (TIF) [file pone.0153753.s001.tif]

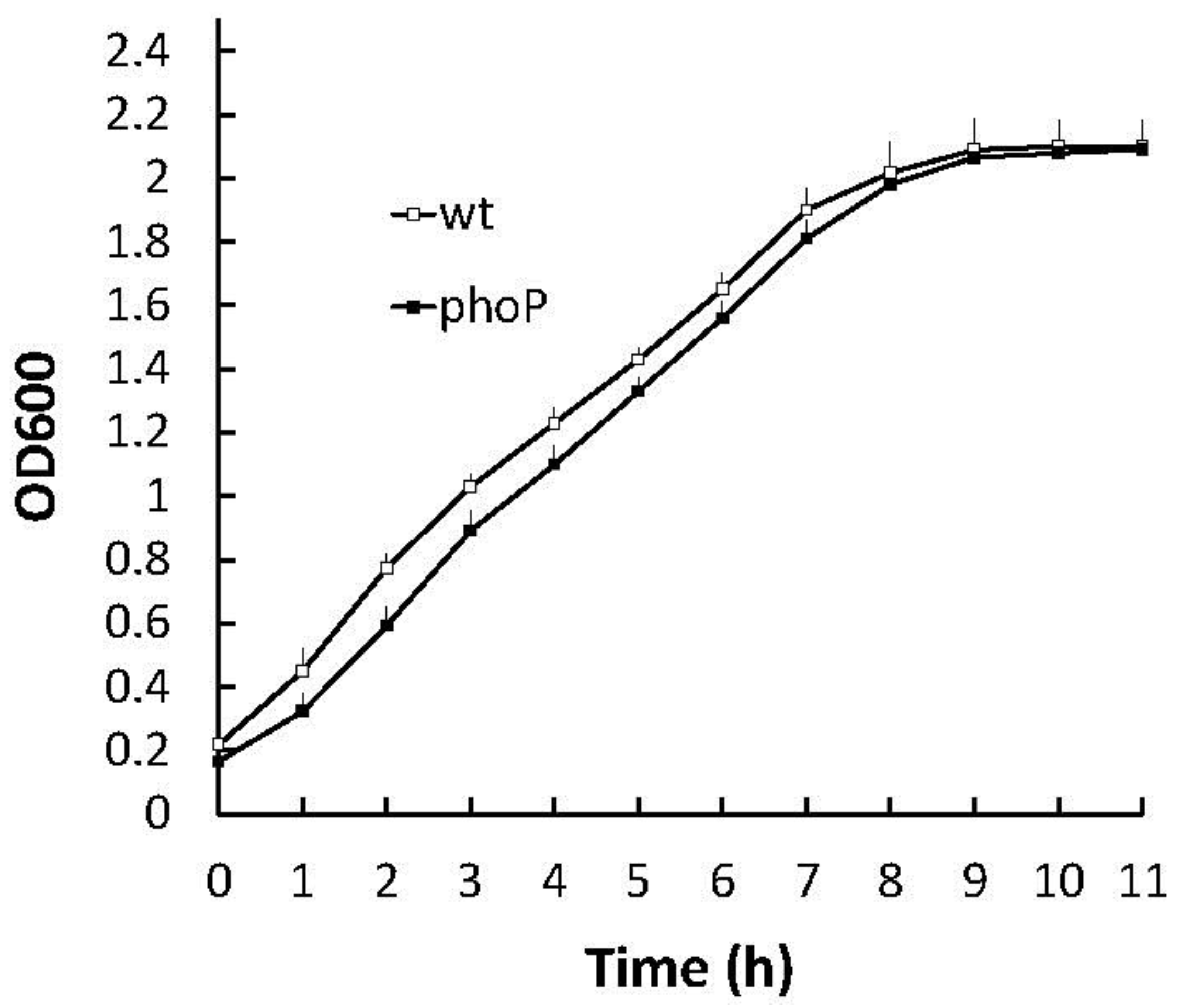

Supplement: S2 Fig — The bacterial growth was expressed as the optical density at 600 nm (OD600). Overnight bacterial cultures of wild-type (wt) and phoP mutant (phoP) were diluted and regrown to the optical density of around 0.2 (OD600) and the growth was monitored at 1-h intervals. The data represent the averages and standard deviations of three independent experiments. (TIF) [file pone.0153753.s002.TIF]
