# Supplementary material for: Stenotrophomonas maltophilia PhoP, a Two-Component Response Regulator, Involved in Antimicrobial Susceptibilities
Source: PLoS One. 2016 May 9;11(5):e0153753. doi: 10.1371/journal.pone.0153753 (PMC4861329; doi:10.1371/journal.pone.0153753)
Supplement: S1 Table — (DOCX) [file pone.0153753.s003.docx]

**S1 Table. Bacterial strains and plasmids used in this study.**

| Strains or plasmid | Genotype or relevant phenotype | Source or reference |
| --- | --- | --- |
| *Stenotrophomonas moltophilia* | | |
| wt | Wild-type (S22) | Clinical isolate |
| phoP | wt derivative; *phoP*-knockout mutant | This study |
| smeZ | wt derivative; *smeZ*-knockout mutant | This study |
| phoPc | *phoP* mutant containing pRK415-phoPQ; PhoP-complemented strain; Tc^r^ | This study |
| *E. coli* | | |
| DH5α | *fhuA2 lac*(*del*)*U169 phoA glnV44 W80’ lacZ*(*del*)*M15 gyrA96 recA1 relA1 endA1 thi-1 hsdR17* | Invitrogen |
| S17-1 λ pir | λ *pir* lysogen of S17-1 [*thi pro hsdR2*  *hsdM+ recA* RP4 2-Tc::Mu-Km::Tn7 (Tp^r^ Sm^r^)]; permissive host able to transfer suicide plasmids requiring the Pir protein by conjugation to recipient cells | [40] |
| Plasmids | | |
| pGEM®-T Easy | High-copy TA cloning vector; Amp^r^ | Promega |
| pEX18Tc | Low copy number plasmid; For gene knockout; Tc^r^ | [19] |
| pRK415 | Low copy number plasmid; Tc^r^ | [19] |
| pRK415-phoPQ  pRK415-phoP | pRK415 containing intact *phoPQ*  sequence including its ribosome binding site  pRK415 containing intact *phoP*  sequence including its ribosome binding site | This study  This study |
