# Supplementary material for: Stenotrophomonas maltophilia PhoP, a Two-Component Response Regulator, Involved in Antimicrobial Susceptibilities
Source: PLoS One. 2016 May 9;11(5):e0153753. doi: 10.1371/journal.pone.0153753 (PMC4861329; doi:10.1371/journal.pone.0153753)
Supplement: S2 Table — (DOCX) [file pone.0153753.s004.docx]

**S2 Table. Primers used in this study.**

| Primers | Sequence (5’ to 3’) | Description |
| --- | --- | --- |
| PhoPQout down1022-R | AGTGGGATGACGAACTGATTGG | For *phoP* gene knockout. Paired with ‘‘PhoPQout down1022-F’’ |
| PhoPQout down1022-F | tctagaTAAACCTTCGCAATCGGTAGTG |  |
| PhoPQout up971-R | tctagaCCAGAAGGATACGCATGAACTC | For *phoP* gene knockout. Paired with ‘‘PhoPQout up971-F |
| PhoPQout up971-F | TATCACGACCCCTATCTGCTGC |  |
| PhoPQcomplementation-F | ctgcagAGCGTGGCAACCGTGGCGAC | For *phoP* complementation. Paired with ‘‘PhoPQcomplementation-R” |
| PhoPQcomplementation-R | ggtaccTTACGGCCCCGGCGGCA |  |
| *smeZ* realtime-F | TGTCCAGCGTCAAGCACC | For *smeZ* real time PCR. Paired with ‘‘*smeZ* realtime-R” |
| *smeZ* realtime-R | GCCGACCAGCATCAGGAAG |  |
| *phoP* realtime-F | GCAATGTCGACCTGACCAG | For *phoP* real time PCR. Paired with ‘‘*phoP* realtime-R” |
| *phoP* realtime-R | GCGGCCGATGAAGACCT |  |
| 16srDNA realtime-F | GACCTTGCGCGATTGAATG | For real time PCR. Paired with “16srDNA realtime-R” |
| 16srDNA realtime-R | CGGATCGTCGCCTTGGT |  |
| *smeZ* EMSA-F | TTG TCCAGGTCCTGGTCC | For *smeZ* EMSA. Paired with “*smeZ* EMSA-R” |
| *smeZ* EMSA-R | CGGAGTCCTGAAGGTTCT CAT |  |
| SmeZout down-F | tctagaGGCGAAGAACGCGATCCTCATCGT | For *smeZ* gene knockout. Paired with ‘‘SmeZout down-R” |
| SmeZout down-R | aagcttGAACTACACGCATGGCTCGACTCC |  |
| SmeZout up-F | ggatccCGCCAGCTCGACCTGCGCTATGC | For *smeZ* gene knockout. Paired with ‘‘SmeZout up-R” |
| SmeZout up-R | tctagaGCCAGGATGATGAAGATCGCCAGC |  |
| PhoPprotein-F | ggatccATGCGTATCCTTCTGGTCGAAG | For PhoP preparation in EMSA. Paired with ‘‘PhoPprotein-R” |
| PhoPprotein-R | ctcgagTCAGCCCTCGTTGCGCGGGAT |  |
| phoPpromoterF | gagctcAGCGTGGCAACCGTGGCGAC | For *phoP* reporter assay and EMSA. Paired with “phoPpromoterR” |
| phoPpromoterR | ctgcagCCAGAAGGATACGCATGAACTC |  |
| M13F | CACGACGTTGTAAAACG AC | M13 primers labelled with IRDye or not for EMSA. Paired with “M13R” |
| M13R | GGATAACAATT TCACAC AGG |  |

The lowercase indicates the restriction enzyme site attached.
